# Forebrain E-I balance controlled in cognition through coordinated inhibition and inhibitory transcriptome mechanism

**DOI:** 10.3389/fncel.2023.1114037

**Published:** 2023-02-24

**Authors:** Tian Tian, You Cai, Xin Qin, Jiangang Wang, Yali Wang, Xin Yang

**Affiliations:** ^1^Shenzhen Key Laboratory of Translational Research for Brain Diseases, Shenzhen Institute of Advanced Technology, Chinese Academy of Sciences, Shenzhen, China; ^2^Department of Neurology, Shenzhen Institute of Translational Medicine, Shenzhen Second People’s Hospital, The First Affiliated Hospital of Shenzhen University, Shenzhen, China; ^3^Department of Medicine, Djavad Mowafaghian Centre for Brain Health, The University of British Columbia, Vancouver, BC, Canada; ^4^Henan International Joint Laboratory of Non-Invasive Neuromodulation, Department of Physiology and Pathophysiology, Xinxiang Medical University, Xinxiang, China

**Keywords:** homeostasis, GABA, forebrain, cognition, E-I balance significance statement

## Abstract

**Introduction:**

Forebrain neural networks are vital for cognitive functioning, and their excitatory-inhibitory (E-I) balance is governed by neural homeostasis. However, the homeostatic control strategies and transcriptomic mechanisms that maintain forebrain E-I balance and optimal cognition remain unclear.

**Methods:**

We used patch-clamp and RNA sequencing to investigate the patterns of neural network homeostasis with suppressing forebrain excitatory neural activity and spatial training.

**Results:**

We found that inhibitory transmission and receptor transcription were reduced in tamoxifen-inducible Kir2.1 conditional knock-in mice. In contrast, spatial training increased inhibitory synaptic connections and the transcription of inhibitory receptors.

**Discussion:**

Our study provides significant evidence that inhibitory systems play a critical role in the homeostatic control of the E-I balance in the forebrain during cognitive training and E-I rebalance, and we have provided insights into multiple gene candidates for cognition-related homeostasis in the forebrain.

## Introduction

Cognitive health is essential for human and animal survival. Cognitive dysfunction is a core symptom in many neurological disorders, including Alzheimer’s disease (AD) (Yang et al., 2018) and schizophrenia (SZ) (Bhat et al., 2021). Excitation-inhibition (E-I) balance in the hippocampus and cerebral cortex is essential for normal cognition (Vogels et al., 2011; Yu et al., 2014; Barron et al., 2016). A functional balance between excitatory and inhibitory synapses (E-I balance) is established and maintained throughout life (Turrigiano and Nelson, 2004). Among them, excitatory synaptic transmission is driven mainly by glutamatergic synapses, whereas inhibitory synaptic transmission involves GABAergic and glycinergic signaling.

A major function of homeostasis is to regulate neuronal activity in a negative feedback manner, thus playing an important role in maintaining forebrain E-I balance (Gilbert et al., 2016) and neuronal activity at the appropriate cognitive level in the ever-changing world (Huber, 2018). At the individual synaptic level, the E-I ratio of neural inputs can be locally regulated by plasticity (Eichler and Meier, 2008). At the neuronal level, E-I balance is globally controlled *via* an excitable threshold and is homeostatically regulated by glutamatergic and GABAergic transmission (Cagetti et al., 2004; Eichler and Meier, 2008). Furthermore, experience or the environment shapes the forebrain E-I balance through homeostatic regulation (Bateup et al., 2013; Herstel and Wierenga, 2021). For example, brief (2–3 days) deprivation of the vision of one eye in rodents (monocular deprivation) reduces network activity (Miska et al., 2018) and improves the acuity of the non-deprived pathway (Fischer et al., 2007). However, the responses of forebrain E-I balance and transcriptome to cognitive experience are not well understood.

Inwardly rectified potassium channel 2.1 (Kir2.1) was wide used to study synaptic and neuronal homeostatic compensative mechanism. Overexpression of Kir2.1 causes hyperpolarization to inhibit neural excitation (Okada and Matsuda, 2008) and network balance control (Paradis et al., 2001). Adult-onset expression of Kir2.1 could induce homeostatic plasticity and presynaptic transcriptional changes in the fruit fly brain (Harrell et al., 2021). Silencing pyramidal neurons with Kir2.1, after synapse formation, causes a homeostatic increase in synaptic inputs to stabilizes network activity (Burrone et al., 2002; Turrigiano, 2011). However, homeostatic regulation of E-I balance in the hippocampus when Kir2.1 is specifically overexpressed in forebrain excitatory neurons remains unknown. Furthermore, the pattern of this rebalance of neural activity in response to subsequent spatial training or environmental changes remains unclear.

Here, we assessed the homeostatic regulation of E-I balance in the forebrain at the electrophysiological and transcriptomic levels by spatial training and overexpressing of Kir2.1 in forebrain excitatory neurons. We also indicated that inhibitory transmission and inhibitory transcriptome mechanisms are important for the homeostatic control of forebrain E-I balance and cognitive experience. This study may provide a reference for understanding the homeostatic control of specific types of neuronal activity abnormalities in diseases including AD and SZ and also suggests a number of potential candidate genes related to neural homeostasis control and cognition.

## Materials and methods

### Animals

All animal experiments were performed according to the protocol approved by the Institutional Animal Care and Use Committee of Shenzhen Institute of Advanced Technology, Chinese Academy of Sciences. Adult (90 ± 2 days old) male C57BL/6 mice were used in this study.

### Generation of the Kir2.1 mutant mice

**FIGURE 1:**
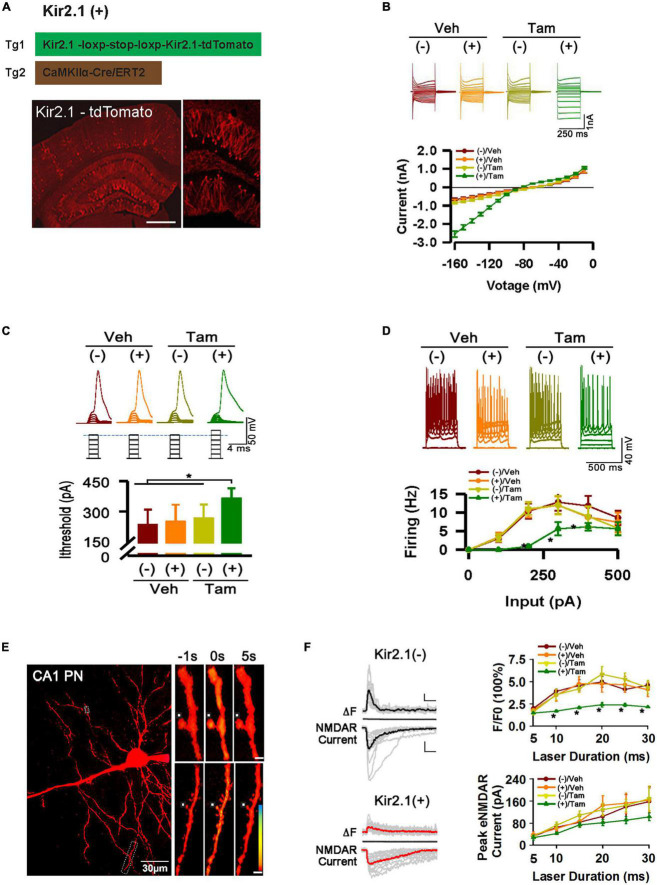
Inhibition of forebrain excitatory neural activity in Kir2.1 knock-in mice. **(A)** A strategy for inducible expression of the Kir2.1 channel (Tg1) in the forebrain neurons by crossing a Tg1 line with a Cre/ERT2 transgenic line under control of CaMKIIα promoter (Tg2), and representative fluorescence images of Kir2.1-tdTomato protein in a sagittal section from the Kir2.1 (+) mice. Bar = 0.5 mm **(B)**. The Kir2.1 currents are plotted against the holding potentials in CA1 pyramidal neurons from the Kir2.1 (+) and the Kir2.1 (–) mice. Traces above the plot are the example recordings from the individual mice. Data are mean ± SEM (*n* = 16 recordings/8 mice per group, ANOVA*p < 0.01). **(C)** The CA1 pyramidal neurons in the Kir2.1 (+) mice require more currents to elicit firing with a 2 ms current step. Top, examples of sub-threshold membrane depolarization and the first action potential elicited by current injection. Square pulses illustrate increasing current injection. Bottom, a bar graph shows the threshold currents required for action potential firing in CA1 pyramidal neurons from the Kir2.1 (+) and the Kir2.1 (–) mice. Data are mean ± SEM (*n* = 12 recordings/6 mice per group, ANOVA *p < 0.01). **(D)** Firing frequency is reduced in the Kir2.1 (+) mice. Top, examples of the CA1 pyramidal neuron firing trains in response to current injection. Bottom, firing frequency is plotted against current injection (500 ms duration, 0 to 500 pA, 50 pA steps). Data are mean ± SEM (*n* = 12 recordings/6 mice per group, ANOVA*p < 0.01). **(E)** Morphological features of a recorded CA1 pyramidal neuron filled with 50 μM Fluo-5F. Box indicates a dendritic segment used for Ca^2+^ image. Ca^2+^ transient in the dendrite was induced by glutamate uncaging. Each image is an average of 6–8 frames taken 1 s before and 5 s after glutamate uncaging (0 s), as indicated. **(F)** Reduction in the peak amplitudes of the dendritic spine Ca^2+^ transients in the Kir2.1 (+) mice. Representatives are the individual traces (gray lines) and averaged responses (black and red lines) recorded in the Kir2.1 (–) mice and Kir2.1 (+) mice. Time courses of fluorescence changes and the NMDA receptor-mediated currents induced by glutamate uncaging recorded in the slices from the Kir2.1 (+) and the Kir2.1 (–) mice. Data are mean ± SEM (*n* = 12 recordings/6 mice per group, ANOVA*p < 0.01). Bar: 200% and 50 pA. In this Figure, the experiments were performed in the Kir2.1 (+) and the Kir2.1 (–) mice 2 days after 5 consecutive days of vehicle or tamoxifen administration.

The Rosa-CAG-Flag-Kir2.1-2A-tdTomato-WPRE targeting vector (Figure 1A) was designed with a CMV-IE enhancer/chicken-actin/rabbit β-globin hybrid promoter (CAG), FRT site, loxp-flanked STOP cassette (with stop codons in all three reading frames and a triple poly(A) signal), Flag-Kir2.1-2A-tdTomato sequence, woodchuck hepatitis virus post-transcriptional regulatory element (WPRE, to enhance mRNA transcript stability), a poly(A) signal, and attB/attP-flanked PGK-FRT-Neo-poly-A cassette. The entire construct was inserted between exons 1 and 2 of the Gt (ROSA)26Sor locus *via* electroporation into C57BL/6-derived embryonic stem (ES) cells. Targeted ES cells were selected and injected into C57BL/6 blastocysts, and chimeric animals were bred into C57BL/6 mice to generate Kir2.1loxp/loxp mutant mice. The conditional mutant strain of theKir2.1 mice was developed by crossing Kir2.1loxp/loxp mice with Tg (CaMK2α-Cre/ERT2) mice (Stock number: 012362, Jackson Laboratory, Bar Harbor, Maine, USA). Male adult (90 ± 2 days old of age) Kir2.1 (+) mice and their Kir2.1 (–) littermates were treated with tamoxifen (Tam) dissolved in core oil (100 mg/kg, i.p., once per day for 5 consecutive days). After recovery for 2 days, the animals were used for the following experiments.

### Morris water maze and open field

The water maze task was performed with a circular tank (120 cm diameter) filled with opaque water (21–23°C) and a hidden platform (6 cm diameter) submerged 1 cm below the surface of the water. The devices (tank and platform) and software [WMT-100] were purchased from Tai Meng Technology Co., Ltd. (Chengdu, China). Before the start of the training trials, the mice were allowed to acclimate to the testing room for 30 min. Mice were trained to find the invisible platform within 70 s on six consecutive days, with four trials per day. If a mouse failed to find the platform within 70 s, it was guided to find the platform and allowed to remain there for 15 s. Escape latency to find the hidden platform, path length, and swim velocity were recorded. After 2 days of rest, the platform was removed, and the mouse was allowed to search for the pool for 70 s (probe tests). The time spent in each quadrant was analyzed. No training means no learning and probe process in MWM, but the animals still swim in the water, while training means an intact MWM experiment. And mice sacrificed at day 6 after probe test.

Locomotion activity was measured in clear boxes measuring 42 cm × 42 cm, outfitted with photo-beam detectors for monitoring horizontal and vertical activity. Data were collected *via* a PC. Mice were placed in a corner of the open-field apparatus and left to move freely. Mice were not exposed to the chamber before testing. Data were individually recorded for each animal during 30 min.

### Electrophysiological recordings *in vitro*

As described previously (Gilbert et al., 2016; Yang et al., 2018), slices (350 μm) of the hippocampus were obtained from male mice at 90 days of age and were placed in a holding chamber for at least 1 h, and saturated with 95 and 5% CO_2_. Artificial cerebrospinal fluid (ACSF, in mM): 124 NaCl, 3 KCl, 1.25 NaH_2_PO_4_, 2 MgCl_2_, 2 CaCl_2_, 26 NaHCO_3_, and 10 glucose. The internal solution contained (in mM): 140 potassium gluconate, 10 HEPES, 0.2 EGTA, 2 NaCl, 2 MgATP, and 0.3 NaGTP, 5 QX-314was used for sEPSCs or mEPSCs, and 140 CsCl, 10 HEPES, 0.2 EGTA, 2 NaCl, 2 MgATP, and 0.3 NaGTP, 5 QX-314 was used for sIPSCs or mIPSCs. The temperature for the patch clamp recording is maintained at about 30 degrees, the electrode enters the liquid surface and corrects the liquid junction potential, and the cell with a resistance less than 20 MΩ after breaking through the cell membrane is considered to be usable. The current-voltage relations for transfected and control neurons were calculated by recording whole-cell currents under a voltage clamp. Voltage steps in 10 mV increments were applied every 250 ms. To measure the amount of current required to reach the action potential threshold, a series of current steps (2 ms or 500 ms duration, 0–1,000 pA range with 100 pA step increments) were injected into the cell until an action potential was generated. Under voltage clamp, the patched cell was held at −70 mV. A bipolar stimulating electrode (FHC Inc., Bowdoin, ME, USA) was placed in the Schaffer collaterals to deliver the stimuli, and the evoked excitatory postsynaptic currents (EPSCs) or inhibitory postsynaptic currents (IPSCs) were recorded under 30 μM bicuculline (Tocris Bioscience, Bristol, UK) or 10 μM CNQX (Tocris Bioscience, Bristol, UK), respectively. Without TTX (Tocris Bioscience, Bristol, UK), spontaneous EPSCs or spontaneous IPSCs were recorded with an external solution containing 30 μM bicuculline or 10 μM CNQX (Tocris Bioscience, Bristol, UK). Miniature EPSCs or miniature IPSCs were recorded with an external solution containing 1 μM TTX, 30 μM bicuculline (Tocris Bioscience, Bristol, UK), or 10 μM CNQX (Tocris Bioscience, Bristol, UK). The synaptic currents were monitored using an Axon 200 B amplifier (Molecular Devices). Spontaneous events were handpicked and analyzed using the Clampfit 10.2 software (Molecular Devices, Sunnyvale, CA, USA), using template matching and a threshold of 5 pA. All data were acquired at 10 kHz and filtered using a low-pass filter at 2 kHz.

### Ca^2+^ imaging

Uncaging of MNI-glutamate, calcium imaging, and whole-cell recordings were performed under a DIC/fluorescence Olympus microscope (FV1000-BX61WI). Slices with a thickness of 300 μm were prepared as described above. The slices were kept at room temperature for at least 1.5 h before being transferred to the recording chamber. Twenty ml of oxygenated magnesium-free ACSF containing 0.2 mM caged glutamate (Tocris Bioscience, Bristol, UK), 1 μM TTX, 30 μM bicuculline, and 10 μM CNQX at 30°C was perfused into the slice recording chamber through a custom-designed flow system driven by pressurized 95% O_2_–5% CO_2_ at roughly 2 ml/min. Slices were examined under a 20 × objective for proper targeting of tdTomato-expressed CA1 pyramidal neurons. To target whole-cell recordings, cells were visualized at a high magnification (60 × objective, 1.0 NA; LUMPLFLN60XW, Olympus). Kir2.1-positive neurons, which were selected on the basis of their pyramidal somata detected under DIC, were RFP-positive on the monitor. Patch pipettes (3–5 MΩ resistance) made of borosilicate glass were filled with an internal solution containing (in mM): 140 potassium gluconate, 10 HEPES, 2 NaCl, 2 MgATP, and 0.3 aGTP, with 20 μM Alexa594 and 50 μM Fluo-5F. For calcium imaging, the neurons were filled *via* the patch electrode for 10–20 min before imaging. The dendrites of the recorded neurons were 30–60 μm below the surface of the slice. Laser scanning and photo stimulation were performed using a 60 × objective lens. One laser was tuned to 405 nm (FV5-LD405-2) to activate MNI-glutamate. The second was tuned to 800 nm (Ti: sapphire laser; 100 fs pulses; Mai Tai HP DeepSee, Spectra-Physics) for the excitation of Alexa Fluor-594 and Fluo-5F. Calcium signals were used to locate dendritic spines 30–80 μm from the soma. The selected dendrite was scanned repeatedly with a series of 200 scans (scan dimensions: 30 μm × 30 μm, 5 μm × 5 μm; 100 ms per scan). The uncaging laser spot was located ∼1 μm away from the spine in Figure 1E. In the first scan, acquisition of NMDAR currents (Axon200B and FV10-ANALOG in our BX61WI microscope) and calcium signals began, and in the 10th frame, uncaging occurred with a constant area 0.25 μm^2^. Glutamate was uncaged using 5–30 ms pulses to generate the I-O curve of the laser strength calcium-NMDAR current under voltage clamp (Vh = −70 mV). To ensure that uncaging was performed in a constant location, image drift was corrected before each line scan acquisition by collecting a frame scan and calculating the cross-correlation to a reference image region of interest (ROI) analysis from these scans, as explained below. Fluorescence changes were analyzed off-line with laboratory-written software using the IGOR-Pro programming environment (Wavemetrics). To study the time course and amplitude of Ca^2+^ rise, the average fluorescence was measured in small “ROIs” (2.25-4 μm^2^) and converted to the percentage change in fluorescence: $\frac{\Delta \,F}{F\,o}=100\times \left(F-F\,r\right)/\left(F\,r-B\right)$, where F is the measured fluorescence signal at any given time, Fr is the average fluorescence from the scans preceding the stimuli, and B is the average value of the background fluorescence in the scanned field that does not contain any part of the dye-filled cell.

### RNA-sequencing and data analysis

Total RNA from the hippocampus and cortex was purified using the RNeasy Plus Micro Kit (Qiagen, Hilden, Germany). RNA quality (RIN 8–9) and quantity were analyzed on a 2,100 Bioanalyzer (Agilent Technologies, Palo Alto, CA, USA) using RNA 6,000 Pico chips; ds-cDNA was produced using the Ovation RNA-seq system V2 (NuGEN, San Carlos, CA, USA) and fragmented using a Covaris S-Series System (Covaris, Woburn, MA, USA). DNA fragments in the 150–300 bp size range were recovered to construct a sequencing library using the Encore NGS Library System I (NuGEN, San Carlos, CA, USA) for 100 bp paired-end RNA-seq using the Illumina HiSeq-2500 sequencer. Raw data were processed using the cutadapt (Martin, 2011) software to remove joints and filter low-quality reads, and 77–121 M clean reads were obtained for each sample with an average Q30 > 95%. RNA-sequencing data alignment and differential gene expression analysis were performed using Tophat2 (Kim et al., 2013) and edgeR (Robinson et al., 2010). Specifically, RNA-sequencing data were aligned to the reference genome (mm10 NCBI buid 38.1) using Tophat2, using default parameters. Uniquely mapped and properly paired alignments were used for further analyses. FeatureCounts (Liao et al., 2013) were used to count the number of reads mapped to each gene. Prior to differential gene expression analysis, for each sequenced library, the read counts were adjusted using the edgeR program package through one scaling normalized factor. Differential expression analysis under the two conditions was performed using the edgeR R package with bcv = 0.1. *P*-values were adjusted using the Benjamini and Hochberg method. A corrected *P*-value of 0.05 and absolute fold change of 2 were set as the thresholds for significantly differential expression. Sample cluster analysis of all detected genes was performed using the pheatmap R package.^1^ Gene Ontology (GO) enrichment analysis of differentially expressed genes was performed using the clusterProfiler R package (Yu et al., 2012; Wu et al., 2021), in which the gene length bias was corrected.

### Fluorescence imaging

As described previously (Shi et al., 2021; Tian et al., 2021), brain tissue was perfused and fixed with 4% paraformaldehyde (PFA) solution, dehydrated with 30% sucrose, and frozen to obtain 30 μm sagittal sections with a cryostat microtome (Leica CM1950). Fluorescence images were obtained using a FV1000-BX61WI microscope.

### Statistical analysis

**FIGURE 2:**
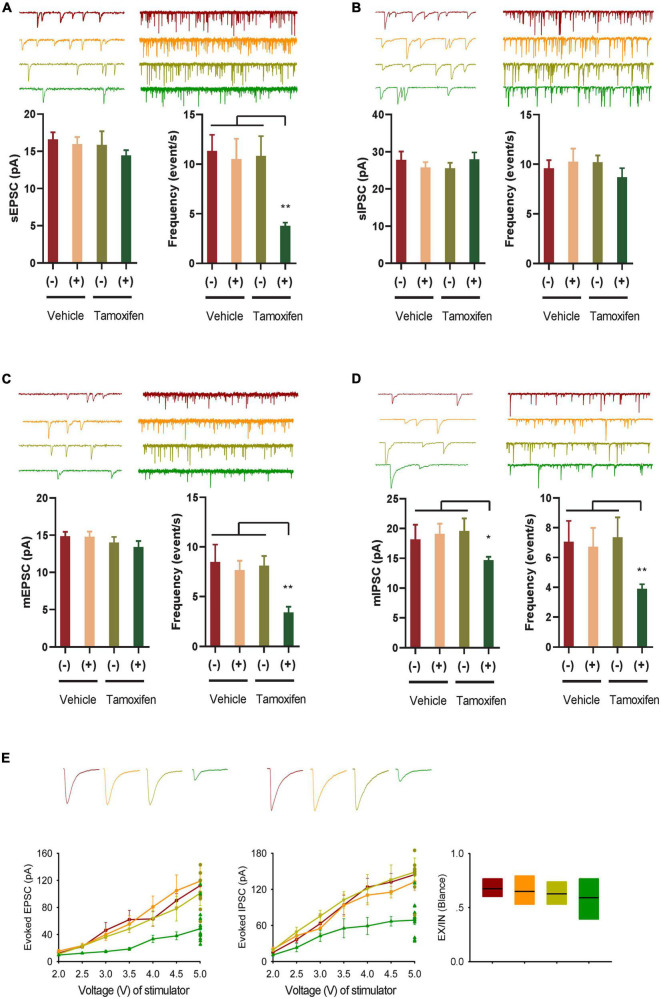
GABA transmission reduction play an important role in E-I rebalance in CA1. **(A,B)** Whole-cell patch clamp recordings from CA1 pyramidal neurons in the slices from the Kir2.1 (–) and the Kir2.1 (+) mice. Representatives and bar graph of the spontaneous excitatory and inhibitory postsynaptic currents (sEPSCs and sIPSCs). Data are mean ± SEM (*n* = 12 recordings/6 mice per group, ANOVA*p < 0.01). **(C,D)** Whole-cell patch clamp recordings from CA1 pyramidal neurons in the slices from the Kir2.1 (–) and the Kir2.1 (+) mice. Representatives and bar graph of the miniature excitatory and inhibitory postsynaptic currents (mEPSCs and mIPSCs). Data are mean ± SEM (*n* = 12 recordings/6 mice per group, ANOVA*p < 0.01). **(E)** The stimulus intensities are plotted against the evoked EPSC and IPSC recorded in the CA1 area of the hippocampus from the Kir2.1 (–) and the Kir2.1 (+) mice. Data are mean ± SEM (*n* = 10 recordings/5 mice per group), and the excitatory divide inhibitory ratio remained comparable between each groups.

**FIGURE 3:**
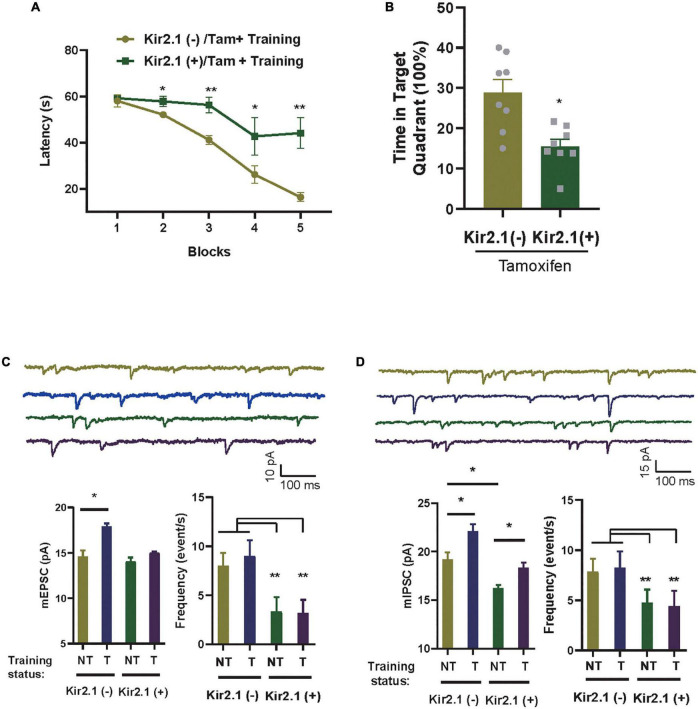
Homeostatic modulation of forebrain E-I balance *via* GABA transmission related to cognitive level and experience. **(A)** The Kir2.1 (+) mice show abnormal performance in a hidden platform version of the Morris water maze. The latency to reach a hidden platform is plotted against the blocks of trials. *N* = 8 mice per group, *t*-test, *p < 0.05 and **p < 0.01. **(B)** The percentage of time spent in search of a hidden platform in the target quadrant during the probe trial, *t*-test, *p < 0.01. **(C,D)** Whole-cell patch clamp recordings from CA1 pyramidal neurons in the slices from the Kir2.1 (–) and the Kir2.1 (+) mice. Representatives and bar graph of the miniature excitatory and inhibitory postsynaptic currents (mEPSCs and mIPSCs). Data are mean ± SEM, (*n* = 10 recordings/4 mice per group, ANOVA, *p < 0.05 and **p < 0.01).

Statistical analysis of the transcriptomics can be found in the RNA-sequencing and data analysis sections. Other statistical analyses were performed using Graphpad Prism version 8. The amount of data collected is described in the corresponding figure legend. The statistical variables in Figures 1–3 are all continuous variables, described using mean and standard deviation, represented as mean ± SEM. In Figures 1, 2, one way ANOVA were used to compare the differences between the Tam (+) group and the other three groups. In Figures 3A, B, an independent samples *t*-test was used to compare the differences between the Kir2.1 (+) and Kir2.1 (-) groups. One-way analysis of variance (ANOVA) was used in Figures 3C, D, and the Bonferroni correction was used for multiple comparisons between different groups. The description of the significance levels of *p*-values is also given in the corresponding figure legends.

## Results

### Inhibition of forebrain excitatory neural activity in Kir2.1 knock-in mice

To study the homeostatic regulation of E-I balance in the forebrain, we genetically suppressed forebrain excitatory neural activity by conditional expression of the inward rectifying potassium channel. Targeting strategies and corresponding genotypic assays are described in detail in the (Section “Materials and Methods). Adult mice received 5 days of tamoxifen (Tam) or vehicle (Veh) treatment, showing successful expression of Kir2.1 channels by morphological and functional confirmation (Figure 1, A–F). First, we showed the presence of a tdTomato-tagged Kir2.1 protein in the forebrain excitatory neurons (Figure 1A). Second, The Kir2.1 currents were plotted against the holding potentials in CA1 pyramidal neurons from Kir2.1 (+) and Kir2.1 (–) mice. The (+)/Tam group mice exhibited large inward rectifying potassium currents (green) recorded from a 250 ms voltage clamp from −160 to −10 mV with Kir2.1 overexpression compared to other groups (Figure 1B). Third, the (+)/Tam group mice had an increased threshold for action potential firing with a 2 ms current step injection compared to the other groups (Figure 1C). The CA1 pyramidal neurons in the Kir2.1 (+) mice required more currents to elicit firing with a 2 ms current step and less firing probabilities *in vitro* with 500 ms current step injection in the (+)/Tam group mice than in others (Figure 1D). Finally, the (+)/Tam group mice showed less calcium influx in Kir2.1 (+) neurons (Figures 1E, F) compared to others with MNI-glutamate uncaging (>10 ms) under a two-photon microscope. Together, these data demonstrate that excitatory neural activity in the forebrain is selectively inhibited by Kir2.1 overexpression.

### GABA transmission reduction play an important role in E-I rebalance in CA1

To study the changes in E-I homeostatic balance after inhibition of forebrain excitatory neural activity, we evaluated the changes in spontaneous and miniature neural transmission in the hippocampus, which is one of the most important areas of the brain for spatial cognition. We observed a reduction in spontaneous EPSCs, but not spontaneous IPSCs in the hippocampal principal neurons (Figures 2A, B). However, the frequency of both miniature mEPSCs and mIPSCs decreased after Kir2.1 overexpression in forebrain pyramidal neurons, and the amplitude of mIPSCs decreased after Kir2.1 overexpression in the forebrain (Figures 2C, D). Furthermore, both evoked IPSCs and evoked EPSCs were reduced after Kir2.1 overexpression in the forebrain of Kir2.1 (+)/Tam group mice compared to those of the other groups, but the E-I currents remained balanced (Figure 2E). These data indicate that reduction in GABA transmission plays an important role in compensating for the E-I imbalance in CA1 elicited by pyramidal neural inhibition.

### Homeostatic modulation of forebrain E-I balance *via* GABA transmission related to cognitive level and experience

In an ever-changing world, experiences regulate E-I homeostatic balance, and rebalanced E-I networks are often thought to optimize learning and memory (Mizumori and Jo, 2013; Huber, 2018; Girardeau and Lopes-dos-Santos, 2021). To study the association between E-I homeostatic balance and cognition, we evaluated the cognitive level in Kir2.1 (+) mice and tested the changes in miniature postsynaptic currents in the hippocampus with or without spatial training. As many of the previous tests showed no differences in neuronal activity-related parameters among the negative control groups, we only examined the parameter changes between Kir2.1 mice with (Kir2.1 (+)) or without tamoxifen (Kir2.1 (–)) induction treatment in the follow-up experiments. First, we showed that compared with Kir2.1 (–) mice, Kir2.1 (+) mice spent more time reaching the escape platform during the 5-day training session (Figure 3A) and less time in the target quadrant during probe testing (Figure 3B), indicating learning and memory impairments in Kir2.1 (+) mice, while no obvious difference in swimming speed in MWM and travel distance in the open field was found between Kir2.1 (–) and Kir2.1 (+) mice (Supplementary Figure 1). Next, we scarified them and compared the miniature postsynaptic currents in trained mice or untrained Kir2.1 (+) and Kir2.1 (–) animals. We found that both excitatory and inhibitory transmission increased after 5-day spatial training in the control group Kir2.1 (–) mice (Figure 3C). However, only GABA transmission, that is, the amplitude of hippocampal mIPSCs, was enhanced after the 5-day spatial training in Kir2.1 (+) mice (Figure 3D). These data indicate that homeostatic modulation of forebrain E-I balance *via* GABA transmission may be related to cognitive level and experience.

### The inhibitory receptor transcriptome plays an important role in homeostatic control of E-I balance and cognitive experience

**FIGURE 4:**
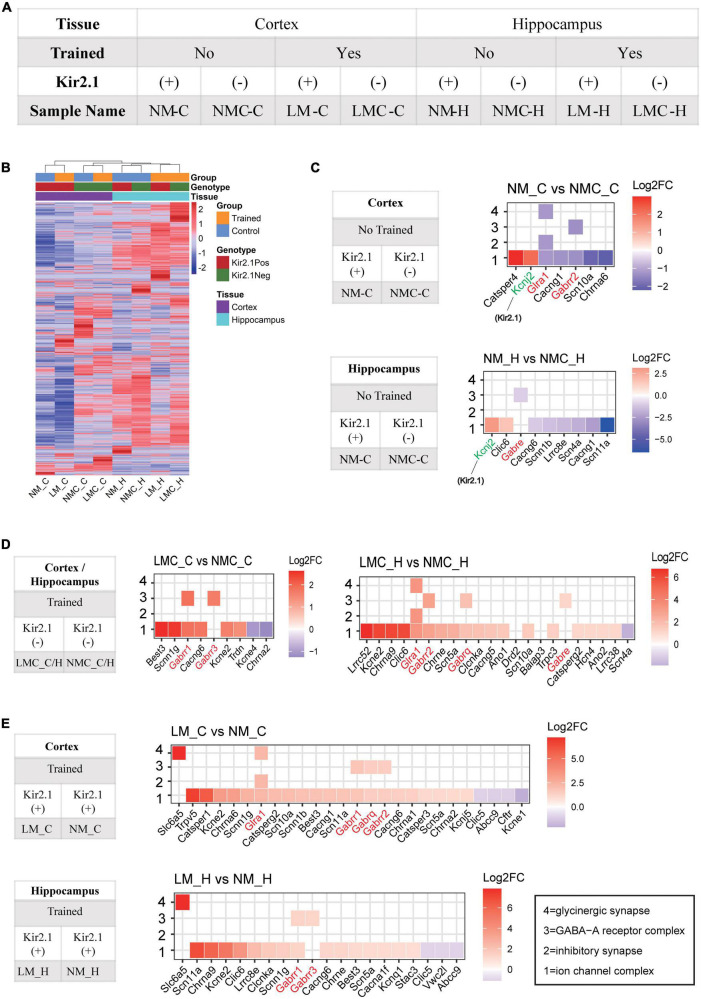
Inhibitory receptor transcriptome plays an important role in homeostatic control of E-I balance and cognitive experience. **(A)** Grouping and naming information for RNA-Seq samples. **(B)** Unsupervised clustering of all gene expression profiles between samples; the distance of the top tree indicates the difference between samples, with the further the distance, the greater the difference. **(C)** Gene ontology analysis of differentially expressed genes in the Kir2.1 (+) mice. **(D,E)** Gene ontology analysis of differentially expressed genes in the spatial training groups. **(C–E)** X axis is sorted by the log_2_ (Fold Change), red represents upregulated, blue represents downregulated, and white is shown around 0. “1” = ion channel complex (GO: 0034702), “2” = inhibitory synapse (GO: 0060077), “3” = GABA-A receptor complex (GO: 1902711), “4” = glycinergic synapse (GO:0098690).

The changes of mIPSC amplitude could be correlated to reduction in inhibitory/GABA receptors in response to cognitive experiences and E-I balance, we determined the underlying genome-wide transcriptome difference in the cortex and hippocampus of trained or untrained Kir2.1 (+)/Kir2.1 (-) mice *via* RNA sequencing. Details of sample grouping and naming, as well as sequencing data, can be found in Figure 4A and Supplementary Table 1; 77–121 M clean reads were obtained for each sample, with an average Q30 > 95%. The count expression matrix for all samples is shown in Supplementary Table 2. Unsupervised clustering of all gene expression profiles between samples showed that spatial training induced more severe transcriptome perturbation in the hippocampus than the overexpression of Kir2.1 in the cortex (Figure 4B). This indicates that spatial training has a more direct effect on the hippocampus, while Kir2.1 overexpression has a more direct effect on the cortex. A detailed list of differentially expressed genes (DEGs) in each group is shown in Supplementary Table 3. From the results of the GO analysis of DEGs caused by Kir2.1 overexpression, we found that the *Kcnj2* gene (*Kcnj2* is the gene symbol name of Kir2.1) was significantly upregulated, this was expected given the fact that they are overexpressing Kir. Hence it served as a control. However, GABA and glycine receptors were significantly downregulated in both the cortex and hippocampus (Figure 4C). However, in the spatial training group, GABA and glycine receptors were significantly upregulated in both the cortex and hippocampus (Figure 4D), particularly in the Kir2.1 (+) group (Figure 4E). This indicates that the transcription mechanism for inhibitory transmission or chloride homeostasis is an important strategy for homeostatic control of the E-I balance in the forebrain network and also suggests many gene candidates for cognition-related homeostasis in the forebrain.

## Discussion

In the present study, we identified that inhibitory systems, including inhibitory transmission and transcription of inhibitory receptors, are important for forebrain E-I balance and cognitive experience, and propose a number of potential genes involved in cognition and homeostasis. This study may provide a reference for understanding the homeostatic control of specific types of neuronal activity abnormalities in diseases, including AD and SZ, and also suggest a number of potential candidate genes related to neural homeostasis control and cognition.

Ion channels play important roles for homeostatic E-I balance. Previously, overexpression of Kir2.1 in forebrain excitatory neurons in mature neurons, inhibited the excitability of excitatory neurons (Burrone et al., 2002) induce homeostatic plasticity (Harrell et al., 2021) and network re-stable (Burrone et al., 2002; Okada and Matsuda, 2008). In this study, we showed a reduction in neural activity in forebrain excitatory neurons lead to the reduction of inhibitory transmission in Kir2.1 (+) mice. Second, in the hippocampus (Figures 4D, E), spatial training increased the transcription of anion and cation channel-related genes, including cholinergic receptors (Chrna9), voltage-dependent sodium and potassium receptor (Kcne2), calcium receptor (Cacng), chloride ions receptor channel (Clcnka), and GABA receptor (Gabrr). The increasing trend of these receptors is not only consistent with the enhancement of synaptic transmission by spatial training, but may also be related to remodeling of cellular ion homeostasis (including cationic sodium and calcium and anionic chloride ions) and the formation of a new E-I balance after spatial training. Furthermore, artificial inhibition of forebrain excitatory neurons in Kir2.1 (+) mice weakened the transcription levels of anion and cation channel-related proteins, including voltage-dependent calcium, GABA, and sodium receptors, which also supported the weakening of inhibitory synaptic transmission and the change in ion homeostasis in Kir2.1 (+) mice. The reduction of inhibitory transmission is consistent with suppressing pyramidal cell activity reduces inhibitory system (such as parvalbumin cells) in the visual cortex from Kir2.1 overexpression mice (Xue et al., 2014). In addition, these ion channels are often important risk factors for diseases with E-I imbalance, suggesting that the cognitive and homeostasis candidate genes identified in this study may also be targets for diseases characterized by E-I imbalance, such as scn-related channels, which have been associated with Dravet syndrome or other epilepsy syndromes (Meisler et al., 2010). It is suggested that influxed/outfluxed anion and cation or related ion channels may serve as a reference for E-I global homeostatic controlled gene compensation in forebrain, which may also be a mechanism in Dravet syndrome or other epilepsy syndromes.

In our study, GABA transmission (mIPSC) homeostatic changes in cognitive experience and E-I balance *via* disinhibition or inhibition enhancement way in direct interneurons-pyramidal neurons or indirect interneurons-interneurons-pyramidal neurons network. Supporting this conclusion, previous studies have suggested that synaptic transmission of GABA can quickly feedback to abnormal neuronal activity homeostasis in the cortex and plays an important role in maintaining E-I balance (Le Roux et al., 2006; Wilhelm and Wenner, 2008). In trained Kir2.1 (+) mice, the amplitude of mIPSC increased but the amplitude of mEPSC is unchanged and frequency still stay lower level in pyramidal neuron in hippocampus, this unchanged mEPSC may result from neutralizing effect of activity inhibition of pyramidal neuron induced by overexpression of Kir2.1 (+) in forebrain excitatory neurons. Furthermore, in the sequencing results, we mainly compared hippocampal data for a single factor, and also tested and analyzed gene transcription (such as GABA receptors) in the cortex, obtaining ion channel changes similar to those in the hippocampus, which improved the problem of low repetition of sequencing data. Interestingly, the inhibitory system is more likely to be disordered in many diseases, and the resulting E-I imbalance is often an important mechanism in these diseases, such as lots of evidence for primary inhibitory dysfunction in autism spectrum disorders (Gilby and O’Brien, 2013; Nelson and Valakh, 2015), less inhibition linked to SZ (Bhat et al., 2021), parvalbumin cells or parvalbumin cells innervated pyramidal neurons pathway selective degenerated lead to E-I imbalance in AD (Yang et al., 2018). Thus, this study suggests the particularity of the inhibitory system in the process of E-I homeostatic balance and cognitive experience, which is of great significance for subsequent studies of the inhibitory system in this type of disease model.

Homeostatic control is essential for adaptation to experience and the accurate regulation of forebrain E-I balance (Turrigiano, 1999; Fong et al., 2015). The forebrain neural networks (Kir2.1 (–) trained) and rebalanced forebrain networks (Kir2.1 (+) trained) may recruit homeostasis to improve the efficiency of cognitive information processing. Although the E-I currents remained in a balanced status in Kir2.1 (+) mice, it recruited evoked IPSCs and mIPSC reduction after Kir2.1 overexpression in the forebrain. And, we suggest that the narrowed neural transmission in Kir2.1 (+) mice may service less bandwidth to processing cognitive information, which supports the impairment of learning and memory in Kir2.1 overexpression mice. Furthermore, spatial training increased neural transmission, such as the amplitude enhancement of mIPSC may be related in part to the increase in transcription of GABAergic, glycinergic and calcium channels related genes after training, which also may increase the bandwidth of cognitive information processing recruited for homeostasis in CA1 from trained Kir2.1 (–) mice compared with that from untrained Kir2.1 (–) mice. Therefore, the level of inhibitory systems (including inhibitory transmission and transcription of inhibitory receptors) that are controlled by homeostasis may be linked to cognitive function.

We did not screen out any significant changes in the transcriptome of excitatory receptor, suggesting that different level of mechanisms homeostatic regulating E-I balance. A transcriptome-level mechanism, which may be more sensitive to global regulation of E-I balance *via* inhibitory system. Another protein-level receptor trafficking mechanism may sensitive to local (such as synapse level) regulation of E-I balance that recruits both excitatory and inhibitory receptors in protein level (Wilhelm and Wenner, 2008; Hou et al., 2011). In this study did not test the total and membrane amounts of glutamate and GABA receptors at the protein level in each group to verify these possible different mechanism. However, we suggested that inhibitory transmission compensation is important for forebrain E-I rebalance and cognitive experience and suggested multiple candidate genes associated with homeostasis and cognitive levels.

## Conclusion

The forebrain excitatory-inhibitory (E-I) homeostatic balance is an important regulator of normal cognition; however, the underlying changes and transcriptomic mechanisms that maintain E-I homeostatic balance and cope with cognitive experiences are largely unknown. This work elucidates that the inhibitory systems, including inhibitory transmission and transcription of inhibitory receptors, are important regulators to forebrain E-I homeostatic balance and cognitive experience, and proposes a number of potential genes involved in cognition and homeostasis.

## Data availability statement

The original contributions presented in the study are publicly available. This data can be found here: https://ngdc.cncb.ac.cn/bioproject/browse/PRJCA013698.

## Ethics statement

The animal study was reviewed and approved by the Institutional Animal Care and Use Committee of Shenzhen Institute of Advanced Technology, Chinese Academy of Sciences (SIAT-IACUC-210303-NS-YXA1694).

## Author contributions

TT was the main investigator in this study. YC analyzed the RNA sequencing data. XQ, JW, and YW revised the manuscript. XY designed the study and wrote the manuscript. All authors contributed to the article and approved the submitted version.

## Footnotes

1
https://cran.r-project.org/web/packages/

## Supplementary material

Supplementary Figure 1 Swimming speed in MWM and travel distance in the open field.
