# Supplementary figures and images for: Forebrain E-I balance controlled in cognition through coordinated inhibition and inhibitory transcriptome mechanism
Source: Front Cell Neurosci. 2023 Feb 24;17:1114037. doi: 10.3389/fncel.2023.1114037 (PMC10000298; doi:10.3389/fncel.2023.1114037)

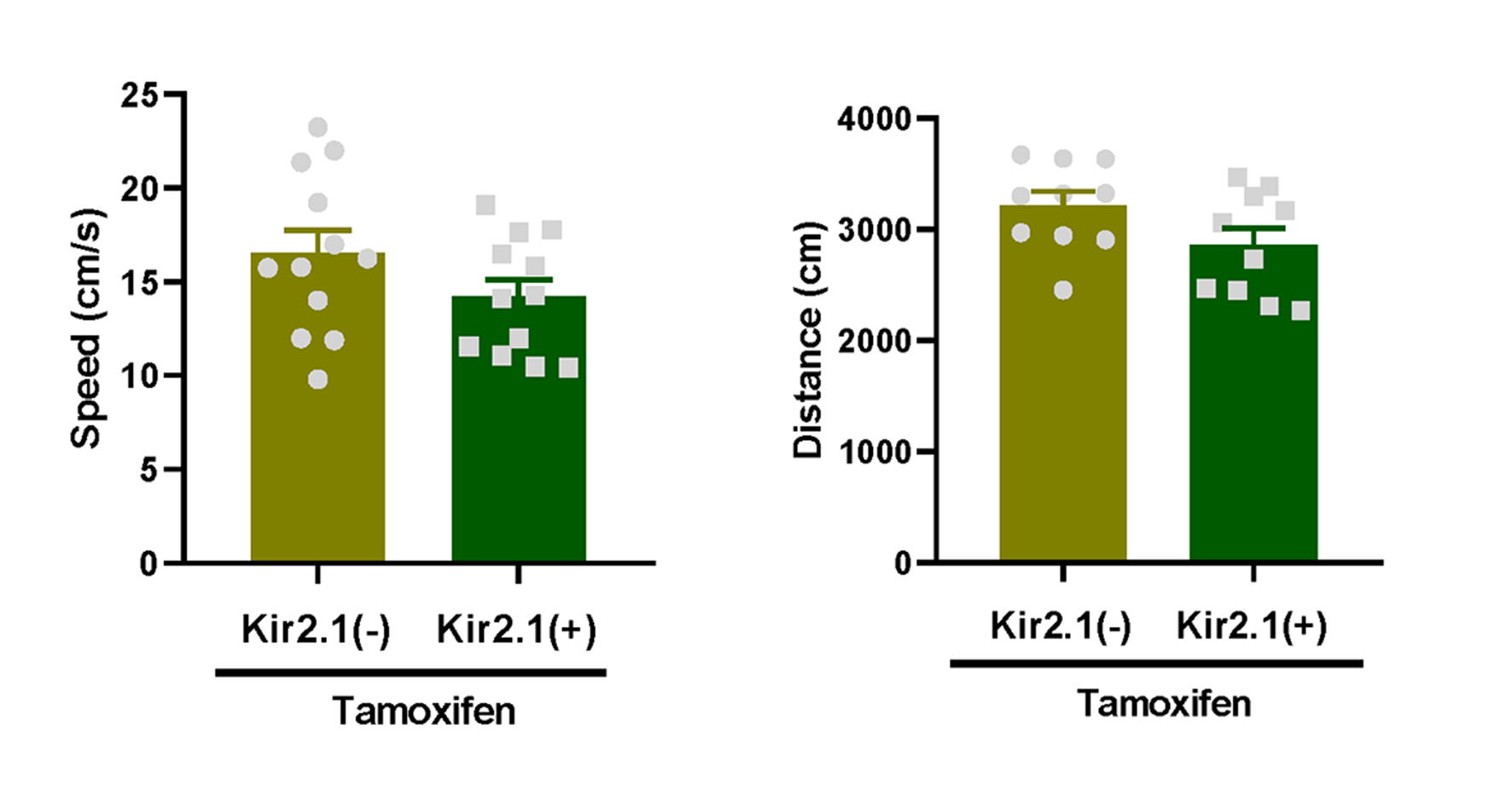

Supplement: Supplementary Figure 1 — Swimming speed in MWM and travel distance in the open field. [file Image_1.tiff]
